# From Protein Engineering to Immobilization: Promising Strategies for the Upgrade of Industrial Enzymes

**DOI:** 10.3390/ijms14011232

**Published:** 2013-01-10

**Authors:** Raushan Kumar Singh, Manish Kumar Tiwari, Ranjitha Singh, Jung-Kul Lee

**Affiliations:** Department of Chemical Engineering, Konkuk University, 1 Hwayang-Dong, Gwangjin-Gu, Seoul 143-701, Korea; E-Mails: singhraushank@gmail.com (R.K.S.); tiwari@konkuk.ac.kr (M.K.T.); ranjita.rsingh@gmail.com (R.S.)

**Keywords:** immobilization, inhibition, protein engineering, selectivity, stability

## Abstract

Enzymes found in nature have been exploited in industry due to their inherent catalytic properties in complex chemical processes under mild experimental and environmental conditions. The desired industrial goal is often difficult to achieve using the native form of the enzyme. Recent developments in protein engineering have revolutionized the development of commercially available enzymes into better industrial catalysts. Protein engineering aims at modifying the sequence of a protein, and hence its structure, to create enzymes with improved functional properties such as stability, specific activity, inhibition by reaction products, and selectivity towards non-natural substrates. Soluble enzymes are often immobilized onto solid insoluble supports to be reused in continuous processes and to facilitate the economical recovery of the enzyme after the reaction without any significant loss to its biochemical properties. Immobilization confers considerable stability towards temperature variations and organic solvents. Multipoint and multisubunit covalent attachments of enzymes on appropriately functionalized supports via linkers provide rigidity to the immobilized enzyme structure, ultimately resulting in improved enzyme stability. Protein engineering and immobilization techniques are sequential and compatible approaches for the improvement of enzyme properties. The present review highlights and summarizes various studies that have aimed to improve the biochemical properties of industrially significant enzymes.

## 1. Introduction

Biocatalysts are extensively used in the industrial production of bulk chemicals and pharmaceuticals, and over 300 processes have already been implemented [1]. In the vast majority of processes, native microbial enzymes of microbial with that exhibit the desired properties are used. Often, for a given biocatalytic process, the native enzyme does not meet the requirements for large-scale application, and its properties thus need to be optimized or modulated. Many industrial enzymes, such as lipases, with a wide range of substrate specificities, are utilized in many processes, often compromising the desired productivity. The role of protein engineering is to overcome the limitations of natural enzymes as biocatalysts and engineer process-specific biocatalysts. This includes optimizing the chemoselectivity, regioselectivity, and, especially, stereoselectivity of the biocatalyst, as well as process-related aspects, such as long-term stability at certain temperatures or pH-values and activity in the presence of high substrate concentrations to achieve maximal productivity. Some improvement in process efficiency can be achieved by modifying the chemical manufacturing process to suit the sensitivities of the biocatalyst (e.g., in terms of pH, temperature, and solvents). The other alternative is to use protein engineering methodologies to generate new biocatalysts to function under more ideal process conditions, followed by immobilization to establish more robust processes. Due to recent advances in protein engineering techniques, numerous examples of the optimization of certain enzyme traits (e.g., thermostability, tolerance towards organic solvents, enantioselectivity) have been reported. This has been achieved both by developing new screening systems and by advancements in the understanding of protein structure. Rational protein design by default variants with very few amino acid exchanges and simultaneous saturation mutagenesis allows the generation of synergistic effects of neighboring mutations. On the other hand, semi-rational approaches have recently been shown to be very efficient in cases where the key amino acids governing the property of interest are known [2]. The choice of method therefore is still a case-to-case decision, depending on the property of interest and existing structural and mechanistic knowledge, as well as on practical considerations, such as the availability of a high-throughput screening or selection system.

**Figure 1:**
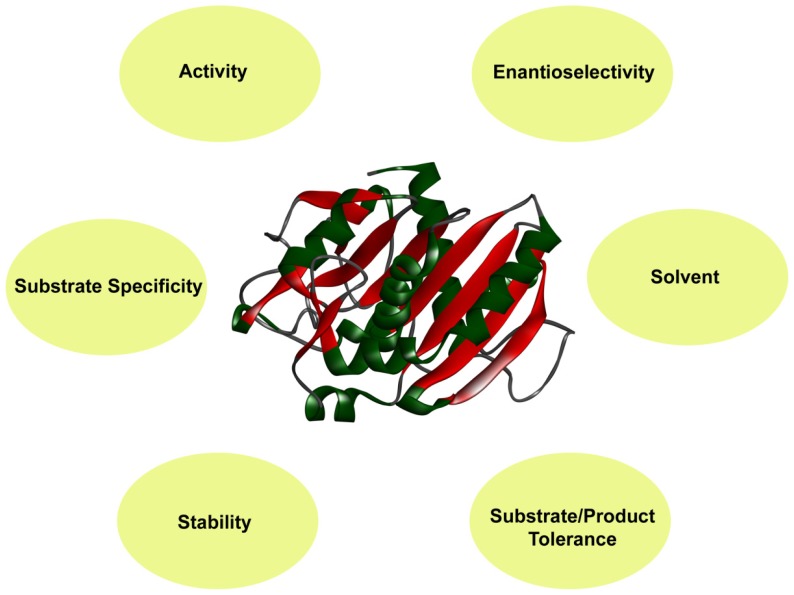
Evolvable enzyme properties for its successful utilization in industrial processes.

Enzymes are considered to be sensitive, unstable at elevated temperatures, and require an aqueous medium for function; these are features that are not ideal for a catalyst, and are undesirable in most syntheses. In many cases a simple way to avoid at least some of these drawbacks is to immobilize enzymes [3]. The immobilization of enzymes has proven particularly valuable and has been exploited over the last four decades to enhance enzyme properties such as activity, stability, and substrate specificity for their successful utilization in industrial processes (Figure 1). In spite of the long history and obvious advantages of enzyme immobilization [4], Straathof *et al.* (2002) estimated that only 20% of biocatalytic processes involve immobilized enzymes [5]. Initially, the main challenge was to find suitable immobilization methods to allow multiple uses of enzymes for the same reaction. With the advancement in immobilization techniques, the focus has shifted to the development of modulated enzymes with the desired properties for certain specific applications. Immobilization has its associated advantages (it allows for multiple, repetitive, or continuous use and has minimum reaction time, high stability, improved process control, multienzyme system, easy product separation, while it is less labor intensive and more cost effective, safe to use, and environmentally friendly) [6] and disadvantages (its lowered activity, conformational change of the enzyme, possibility of enzyme denaturation, changes in properties, mass transfer limitations, and lowered efficacy against insoluble substrates). This review covers different strategies of protein engineering and immobilization to modulate the properties of enzymes to suit industrial processes.

## 2. Protein Engineering to Upgrade Industrial Enzymes

In the past few decades, biocatalysts have been successfully exploited for the synthesis of various complex drug intermediates, speciality chemicals, and even commodity chemicals in the pharmaceutical, chemical, and food industries due to their inherent ability to catalyze reactions with high velocity and unmet specificity under a variety of conditions, as well as their potential as a greener alternative to chemical catalysts. The increasing interest in applying enzymes in industrial and household catalysis has spurred the development of protein engineering methodologies for novel biocatalysts with new or improved properties. Recent advances in recombinant DNA technology, high throughput technology, genomics, and proteomics have fueled the development of new biocatalysts and biocatalytic processes. Since the beginning of large-scale (recombinant) enzyme production for industrial applications, protein engineering has emerged as a powerful tool to improve enzyme properties. Enzymes with the desired properties such as enhanced activity, high thermostabilty, and specificity under industrial conditions can be obtained by optimizing process conditions and by protein engineering (Table 1).

### 2.1. Activity

Improving the activity of an industrial enzyme is often a primary goal. This is partly because naturally available enzymes are usually not optimally suited for many processes in industrial applications. Many industrial enzymes, such cellulases, amylases, lipases, and even proteases, act on insoluble substrates. Therefore, the rate of substrate turnover may be limited by diffusion, and controlled by enzyme mobility at the surface or by on/off enzyme desorption rates [61]. These, in turn, are often related to the surface properties of the enzyme and the conditions at the interface between the enzyme and substrate [62]. A comparative study of the experimental results from several site-directed variants with structural modeling of fungal lipase from *Rhizopuss oryzae* has provided much insight into the molecular mechanism of catalysis [63]. Substitutions at Glu87 and Trp89 in the lid region have been suggested to alter the activity of the lipase from *Humicola lanuginosa* (lipolase) [64].

Cellulases and xylanase have become major focus in recent years due to their ability to provide the soft feel of stone-washed jeans in textile processing, fabric care benefits (such as color crispness) when used in laundry detergents [65], and reduction of the quantity of chemicals required for bleaching in the pulp and paper industry, thereby minimizing environmental impact [66]. Tyr169 in the *Trichoderma reesei* cellobiohydrolase II catalytic domain plays an important role in distorting the glucose ring into a more reactive conformation [67]. A detailed discussion of different families of xylanases and their structure and activity is provided in a review article [68] in *Current Opinion in Biotechnology*.

### 2.2. Thermal Stability

**Figure 2:**
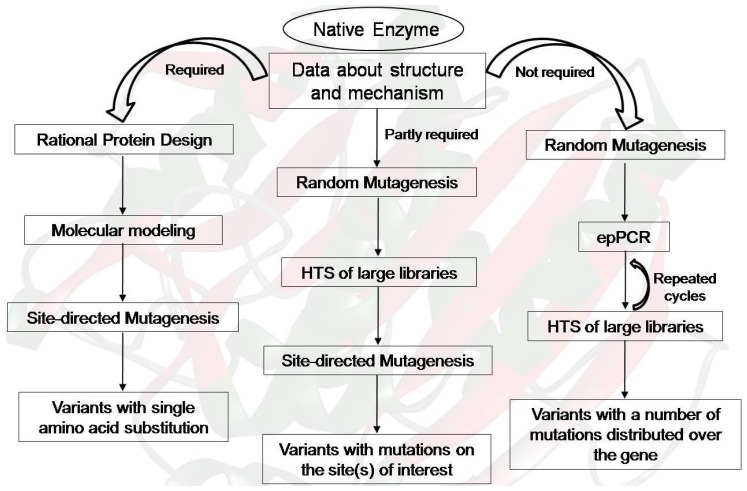
Schematic representation of protein engineering strategies. Engineering method should be selected on the basis of the structural and mechanistic information and the feasibility of a high-throughput screening (HTS) system for screening or selection.

Enhanced thermostability is one of the most common properties desired as output from a protein engineering study and is often an important economic factor. The stability of an enzyme is affected by many factors, such as temperature, pH, solvent, and the presence of surfactants. Among all possible deactivating factors, temperature is the best studied. At elevated temperatures, many enzymes tend to become (partly) unfolded and/or inactivated, meaning that they are no longer able to perform the desired tasks. There are two types of protein stability, thermodynamic and long-term, that are crucial from an applied perspective. Numerous protein engineering strategies have been reported in the last 10 years. Site-directed mutagenesis (SDM) and directed evolution have been exploited to engineer catalysts with improved thermostability (Figure 2). However, a combination of both strategies is becoming popular among researchers. Cherry *et al.* (1999) reported a combination of rational engineering and directed evolution techniques to improve the resistance of a fungal peroxidase towards hydrogen peroxide and high temperature, at high pH, with the aim of making the enzyme better suited for laundry applications [69]. They obtained an enzyme variant with drastically improved thermal stability (200-fold greater than that of the wild-type enzyme) under conditions that mimic those in a washing machine. In another early study, Martin *et al.* (2001) described the stabilization of a cold-shock protein from *B. subtilis* (Bs-Csp) [70]. The size of the library was limited by the transformation efficiency of *Escherichia coli*, but still amounted to a respectable 107 variants. After six rounds of phage selection from 107 variants, five variants displayed increased stability. The most stable variant, displaying a remarkable improvement in T_m_ (a 22 °C increase), differed at six randomized positions from its mesophilic parent. Very few charged residues were found in the selection, which might be related to the use of guanidine hydrochloride (GdmHCl) in the selection scheme. After applying several selection regimes (temperature, amount of protease), mutants displaying up to a 28 °C increase in T_m_ were obtained. The best mutant from the thermal screen differed at all six positions from the wild-type, the thermostable counterpart, Bc-Csp, and from the variant obtained by GdmHCl selection. Palackal *et al.* (2004) reported one of the highest stabilizations ever obtained by enzyme engineering (increase in T_m_ of over 30 °C) [71]. Their starting point was a xylanase that was discovered by screening 50,000 plaques from a complex environmental DNA library derived from a sample of fresh bovine manure. The use of site-saturation mutagenesis and the screening of approximately 70,000 clones led to the identification of nine interesting mutations, which, when combined, increased the Tm by 34.2 °C.

Directed evolution is a powerful engineering method, and it is often used to design enzymes with increased thermostability [72]. By screening for initial activity and residual activity at an elevated temperature, both the thermostability and activity of mesophilic subtilisin E [73], psychrophilic subtilisin S41 [74] and mesophilic *p*-nitrobenzyl esterase [75] were significantly increased using directed evolution strategies. Enzymes that have been improved by directed evolution have already been commercialized [72]. A major advantage of this engineering method over SDM is that no knowledge about enzyme structure is necessary. Since there is still much to be learned about thermostabilization mechanisms, SDM approaches often yield disappointing results. *Bacillus subtilis* subtilisin E was converted into an equivalent of its thermophilic homolog thermitase through the successive application of one round of error-prone PCR, one step of DNA shuffling (to combine the properties of the best variants), and four additional rounds of error-prone (ep) PCR. The evolved enzyme was 15 times more active than subtilisin E at 37 °C, it showed a 16 °C increase in T_opt_, and its T_m_ at 65 °C was more than 200 times that of subtilisin E [73]. In another experiment, the thermostability of *B. subtilis p*-nitrobenzyl esterase was enhanced through five cycles of epPCR followed by one step of DNA shuffling [76]. The evolved esterase showed a 14 °C increase in T_opt_ and a 10 °C increase in T_m_, and it was more active than the wild-type enzyme at any temperature.

Using a directed evolution approach, Oshima and coworkers (2001) obtained four variants of a thermophilic 3-isopropylmalate dehydrogenase with enhanced specific activities at low temperatures [77]. Two of these variants exhibited wild-type thermostability, while the other two exhibited decreased thermostability, with an inverse correlation between activity and stability. A similar result was obtained in case of thermostable *S. cerevisiae* 3-isopropylmalate dehydrogenase variants evolved using genetic selection in an extreme thermophile [78]. On the other hand, mutations targeted at areas whose unfolding is limiting in the protein denaturation process can provide extensive stabilization. Good illustrations can be found in the stability studies of *Bacillus stearothermophilus* thermolysin-like protease. Stabilizing mutations were all located on the surface, around one flexible loop located in the β-pleated *N*-terminal domain [79–81]. The association of eight mutations in the same area resulted in a 340-fold kinetic stabilization of *B. stearothermophilus* thermolysin-like protease at 100 °C and did not affect catalytic activity at 37 °C [82].

Successes in substituting left-handed helical residues with Gly or Asn, in introducing prolines in surface turns or loops, in introducing non-local surface ion pairs, and in creating disulfide bridges that dock loops to the protein surface are well documented [82–86]. Two types of mutations, Gly to Xaa and Xaa to Pro, can be introduced. In the first case, the newly introduced β-carbon should not interfere with neighboring atoms. In the second case, the substitution site should have specific dihedral angles (ϕ and ψ) in the regions −50 to −80 and 120 to 180 or −50 to −70 and −10 to −50, and the residue preceding the potential proline should also have a specific conformation. In addition, the proline ring should not interfere with neighboring atoms, and the substitution should not eliminate stabilizing non-covalent interactions. The most promising strategies for thermostabilization using SDM should focus on the surface areas, particularly loops and turns, and on creating additional non-local ion pairs. The introduction of disulfide bonds, chemical crosslinks, and salt bridges has been widely used to increase stability, although not all disulfide bonds increase stability [87]. Loops can be made more rigid by decreasing their intrinsic entropy of unfolding. There are now several examples of proteins that have been stabilized by the introduction of numerous mutations with cumulative small stabilizing effects (Table 1) [76,88–91]. However, a clear conclusion to be drawn from others is that very large stability differences in some cases are due to only one or a few point mutations [39,45,46,92–94]. Another method is to anchor the loops to the protein surface, either by non-covalent interactions or using a disulfide bridge. Introducing a disulfide bridge in a semiflexible area of the protein should help compensate for any conformational strain created by the disulfide bridge [80].

### 2.3. Solvent Stability

The use of organic solvents as reaction media for biocatalytic reactions has proven to be an extremely useful approach to expand the range and efficiency of the practical applications of biocatalysis [95]. Unfortunately, the majority of naturally available biocatalysts are usually not optimally suited for catalysis in non-aqueous solvents in industrial processes. Polar solvents of practical interest, such as acetone or dimethylformamide (DMF), interact with the enzyme and associated water molecules and drastically reduce catalytic activity [96]. The ability to use enzymes in non-aqueous solvents has increasingly drawn the attention of researchers worldwide to the problems and potential of non-aqueous biocatalysis in chemical transformations that are useful for many industries. There are many potential advantages of enzyme catalysis in non-aqueous solvents, including (1) enhanced solubility of substrates such as lipids and phospholipids; (2) novel chemistry in synthetic applications; (3) altered substrate specificity; (4) easy product recovery; and (5) reduced microbial contamination [95,97]. Despite the many limitations of non-aqueous environments, particularly the poor stability of enzymes in polar organic solvents, research in this area has made tremendous progress in recent years, focusing in particular on the elucidation of enzyme structure and improvement of stability and catalysis in organic solvents, for synthetic applications.

Enzymes may be redesigned to enhance catalysis and stability in non-aqueous solvents by engineering their amino acid sequences, thereby altering their functional properties to adapt the new solvent environment. Many strategies to engineer proteins for non-aqueous environments have been developed [98–102]. Among them, directed evolution and rational design approaches are widely utilized. Directed evolution approaches involving SDM are more efficient when detailed structural information and the molecular basis for the property of interest are poorly understood. Impressive work has been done using an evolutionary approach consisting of multiple steps of random mutagenesis and screening to improve the activity and stability of subtilisin E in high concentrations of organic solvent [99]. Random mutagenesis by PCR techniques combined with screening resulted in enhanced activity in the presence of dimethylformamide (DMF). The triple mutant (D60N + Q103R + N218S) is 38 times more active than wild-type subtilisin E in 85% DMF. Single amino acid substitutions increase the activity and stability of mutant enzyme in mixtures of organic solvents and water, and the effects of these mutations are additive. The N218S substitution is reported to cause improved activity and stability of subtilisin BPN, as well as improved activity and stability of subtilisin E in the presence of DMF. The double mutant Q103R + N218S is 10 times more active than the wild-type enzyme in 20% (*v*/*v*) DMF and twice as stable in 40% DMF. Similar examples of enzyme stabilization also involve subtilisin *E* in polar organic solvents (DMF) by rational engineering. The substitution of Asp248 with three amino acids of increasing hydrophobicity, Asn, Ala, and Leu, resulted in stabilized variants with respect to wild type in 80% DMF. This stabilization was only observed at high concentrations of organic solvent, and not at low organic acid concentrations (40% DMF). In contrast, the mutant N218S stabilizes subtilisin E at both low (40%) and high (80%) concentrations of DMF. The double mutant (D248N + N218S) protein is 3.4 times more stable than the wild type in 80% DMF [103]. This study provides additional evidence that substitution of surface-charged residues is generally additive and useful for stabilizing enzymes in organic solvent. Using the directed approach, random mutagenesis, recombination, and screening, Song and Rhee (2001) obtained three variants of phospholipase A with enhanced stability and activity in organic solvents [104]. Using a similar strategy, Arnold and coworkers (2001) reported a variant of horseradish peroxidase with enhanced stability in the presence of H_2_O_2_, sodium dodecyl sulfate (SDS), and salts [105].

### 2.4. Substrate Specificity

Engineering novel enzyme specificity using a directed evolution approach is of increasing importance to the chemical and pharmaceutical industries. Several recent reports describe significant advances made toward this goal. Using epPCR, followed by saturation mutagenesis and screening, Reetz and coworkers [106,107] considerably modified the enantioselectivity of a *Pseudomonas aeruginosa* lipase towards 2-methyldecanoate from *E* = 1.04 × (2% enantiomeric excess [ee]) to *E* = 25 × (90%–93% ee) (where *E* is the enantioselective factor). None of the five amino acid substitutions in the best variant was located near the substrate-binding pocket [108]. Using a similar approach, Arnold and coworkers (2000) successfully inverted the enantioselectivity of a hydantoinase from D selectivity (40% ee) to moderate L preference (20% ee) [7]. An impressive example of switching enzyme substrate specificities can be seen in the DNA shuffling of two highly homologous triazine hydrolases [109]. The two enzymes, AtzA and TriA, are distinguished by nine amino acids, and hydrolyze s-triazines by dechlorination and deamination, respectively, with little overlap in substrate preference. Permutations of the nine amino acid differences by DNA shuffling resulted in a set of variants that hydrolyzed five of eight triazines that were not substrates for either starting enzyme. The *E. coli*d-2-keto-3-deoxy-6-phosphogluconate (KDPG) aldolase catalyzes a highly specific reversible aldol reaction on d-configurated KDPG substrate. Using directed evolution approach, Wong and coworkers (2000) obtained a variant capable of accepting both d- and l-glyceraldehyde as substrates in a non-phosphorylated form [110]. Notably, all the six substitutions found in the resulting variant were far away from the active site. A double mutant (Lys133Gln/Thr161Lys) of the same enzyme with a considerably altered substrate profile was reported using epPCR and SDM [111]. The directed evolution of oxygenases exhibited similar results [112–115].

### 2.5. A Way Forward: Hybrid Approaches

Over the past decade, advances in DNA technologies and bioinformatics have substantially accelerated the redesign of proteins with novel or desired characteristics. Proteins can be rationally engineered if information about the catalytic mechanism and structure of a protein is known. However, practical experience shows that protein dynamics is complex and it is understood that substitutions distant from the active site can alter the characteristics of a protein [116,117]. Directed evolution would be a more suitable approach for the proteins whose structures and mechanisms are known. However, this method is driven by multiple rounds of selection and screening and each step improves the enzyme. In cases where high-throughput screens are unavailable, a semi-rational method involving site saturation mutagenesis may be applied to identify target residues through computational methods. This way, the library size can be reduced to a manageable size to increase the chance of discovering desired variants.

Recently, molecular modeling has been employed to predict protein structures and various algorithms are being used to predict secondary and tertiary structures based on amino acid sequence [118–120]. This advancement immensely supports rational design of proteins with unknown structures [118,120]. Furthermore, simulation and molecular docking of small molecules to proteins is another field that has advanced tremendously over the years [121,122]. Molecular docking can facilitate understanding of ligand–protein interactions and hence rational designing of proteins for desired properties [123,124].

Assimilation of unnatural amino acids (uAAs) into proteins has opened new possibilities for creating proteins with novel functions and improved properties [125]. Substitution of natural amino acids with a uAA at multiple specific sites in a protein and insertion of uAAs into proteins to expand the genetic code are two different approaches. With such progress, assimilation of uAAs into proteins may soon be a routine practice in the field of protein engineering, and may become a powerful technique for designing novel enzymes to meet the demands of synthetic biology [126].

## 3. Immobilization to Upgrade Industrial Enzymes

**Figure 3:**
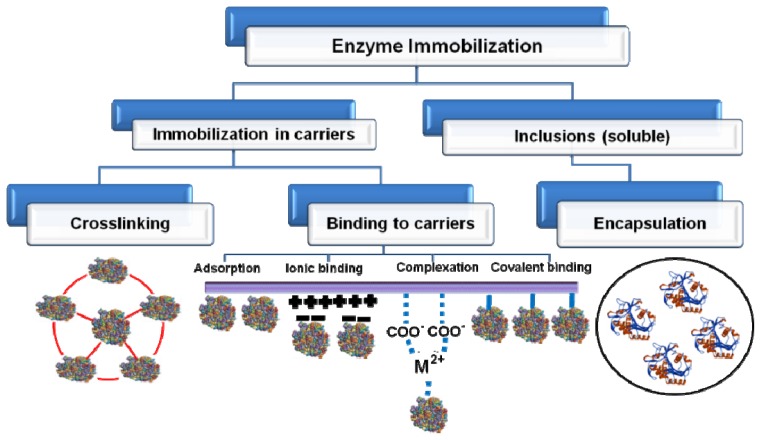
Immobilization of enzyme via different routes.

Since the first industrial application of immobilized amino acylase in 1967 for the resolution of amino acids, enzyme immobilization technology has attracted increasing attention and considerable progress has been made in recent decades. Enzymes are exploited as catalysts in many industrial, biomedical, and analytical processes. There has been considerable interest in the development of enzyme immobilization techniques because immobilized enzymes have enhanced stability compared to soluble enzymes, and can easily be separated from the reaction. Approaches used for the design of immobilized enzymes have become increasingly more rational and are employed to generate improved catalysts for industrial applications. There are a variety of methods used to immobilize enzymes, the three of the most common being adsorption, entrapment, and crosslinking or covalently binding to a support (Figure 3). Recently, the major focus of enzyme immobilization is the development of robust enzymes that are not only active but also stable and selective in organic solvents. The ideal immobilization procedure for a given enzyme is one that permits a high turnover rate of the enzyme while retaining high catalytic activity over time. Proteins are immobilized either by physical adsorption to the surface of the nanoparticle or by covalent bonding to previously functionalized nanoparticles.

### 3.1. Activity

In the early 1960s Goldstein *et al.* observed the enhancement of enzyme activity upon immobilization because of the microenvironment effect [127]. In general, enhancement of enzyme activity upon immobilization depends on the microenvironment, partition effect, diffusion effect, conformational change, molecular orientation, conformational flexibility, conformation induction, and binding mode. It has been observed that many immobilized enzymes exhibit higher activity than the corresponding native enzyme. For example, immobilized lipase was 50-fold more active than the native enzyme [128]. In literature, there are few reports of enzymes that exhibit decreased K_m_ and increased V_max_ upon the formation of crosslinked enzyme aggregates (CLEA) [73,129–131] compared the covalent immobilization and physical adsorption of a cellulase enzyme mixture on plasma immersion ion implantation (PIII)-treated and untreated polystyrene, respectively. Activity on the PIII-treated surface was found to be higher than that on the untreated surface. The activity on the untreated surface may be lower because enzyme aggregates are generally not as active as the same number of unaggregated enzymes. It has been described that immobilized enzymes can be more active than the native enzymes, when the inhibiting effect of the substrate is reduced. For example, the immobilization of invertase from *Candida utilis* on porous cellulose beads led to decreased substrate inhibition and increased activity [132]. Table 2 includes the examples of enzymes with improved activity via immobilization. Recent advancements in immobilization techniques, particularly in oriented immobilization, have generally resulted in higher activity relative to their randomly immobilized counterparts, because of favorable accessibility or avoidance of active site modification [133]. These methods focus on the improvement of immobilized enzyme properties without compromising activity. In case of trivial immobilization techniques, activity retention was often marginal, requiring laborious screening of immobilization conditions such as enzyme loading, pH, carrier, and binding chemistry [134]. Higher enzyme activity can occasionally be achieved, especially for allosteric enzymes such as lipase. Lipases exist in two different conformations [135,136]: the closed (inactive) form with lids covering the active site, and the open form, where the active site is fully exposed to the reaction medium [137–139], and enhancement of enzyme activity relative to that of the native enzyme can be observed. Conformation-controlled activity was found to be strongly dependent on the nature of the support used immobilization. For example, lipase PS (*Pseudomonas cepacia*) immobilized on toyonite, celite, glass, and amberlite, exhibited the highest activity on toyonite (37.2 μmol·min^−1^·mg^−1^) and the lowest on amberlite (0.4 μmol·min^−1^·mg^−1^) [140].

An enzyme immobilized in an oriented manner using a covalent procedure preserves its functionality and leads to the assemblage of a homogeneous layer. It was demonstrated that the lipase from *Mucor risopus* immobilized in organic solvent was more active in organic solvent whereas the same immobilized in an aqueous medium had almost no activity in organic solvents. It might be that the position of binding of the enzymes to the carrier in organic solvents is different from when immobilization is performed in an aqueous medium [159]. Recently, analysis of cytochrome c reductase assay revealed that oriented enzyme samples resulted in about a three-fold higher activity in solution than randomly bound samples because of favorable accessibility [160]. Orderly oriented enzyme molecules generally have higher activity or stability relative to their randomly immobilized counterpart [133].

When the enzyme is covalently immobilized in porous materials using the conventional protocol, diffusion limitations of the enzyme inside the carriers and slow binding reactions between the enzyme and activated carrier groups slow down the covalent immobilization of the enzyme [155]. Activity retention by carrier-bound immobilized enzymes is usually approximately 50% [161]. At high enzyme loading, especially, diffusion limitation might occur as a result of the unequal distribution of the enzyme within a porous carrier, leading to a reduction of pore volume available to the substrate and product diffusion. Bezbradica *et al.* (2009) reported the specific activity of microwave-assisted immobilization of lipase from *Candida rugosa* on Eupergit C250L was higher because of higher diffusion rate [162]. Microwave irradiation technology has been reported by several researchers to overcome the diffusion limitation. For instance, penicillin acylase from *Bacillus megaterium* was covalently immobilized on mesocellular silica foams [155].

It is a common belief that conformational flexibility provides a protein structure with the essential mechanism for carrying enzymatic reactions. The effect of conformational flexibility on the enzyme activity was initially justified by the observation that enzyme immobilized on a carrier via a suitable spacer often resulted in better retention of activity than the enzyme immobilized without a spacer [163–166]. Bigdeli *et al.* (2008) reported the retention of conformational flexibility of glutamate dehydrogenase, used as a model allosteric protein, upon its immobilization on self-assembled monolayers-modified gold [167].

Activity of immobilized enzyme is often influenced by binding mode. The effect of binding mode may be reflected by the number of bonds formed between the carrier and the enzyme molecules, the position of the bonds and the nature of the bonds. Immobilization of β-galactosidase from *E. coli* and *Kluyveromyces lactis* on thiolsulphinate-agarose and glutaraldehyde-agarose clearly demonstrates the relation between the number of bonds formed between the enzyme and the carrier and enzyme activity [168]. These two enzymes are richer in the lysine residues resulting in more bonds formation with glutaraldehyde-agarose and less retention of activity. Interestingly, α-amylase immobilized on thionyl chloride (SOCl_2_) and carbodiimide (CDI) activated poly (Me methacrylate-acrylic acid) microspheres retained 67.5% and 80.4% of activity, respectively [169].

Conformational changes of the enzyme induced by immobilization usually decrease the affinity to the substrate (increase of K_m_). Furthermore, a partial inactivation of all, or the complete inactivation of a part of the enzyme molecules may occur (decrease of V_max_). Hanefeld *et al.* (2008) reported that the difference in kinetics between lipases immobilized on different supports was ascribable to conformational changes induced upon enzyme-polymer interaction [170]. A similar observation had been reported for immobilized invertase on white and black lahar (volcanic mudflow) by the silane–glutarldehyde method [171]. Immobilization brought about an increase in the K_m_ but a decrease in the V_max_ and these changes were correlated to immobilization induced conformational changes in the enzyme [172].

### 3.2. Thermal Stability

Enormous efforts have made by several research groups to enhance the stability of enzymes with the help of immobilization techniques [170], for use in industrial processes, in which the cost-contribution of the immobilized enzyme is often the indicator of process viability [173]. Random immobilization may not always applicable to improve enzyme rigidity; in some cases, the enzyme stability may even be reduced upon immobilization [174]. During random immobilization, the support may establish undesirable interactions with the enzyme resulting in the destabilization of the enzyme structure. Many useful strategies have been developed for enzyme stabilization by immobilization, such as crosslinking, multipoint attachment, and covalent and non-covalent immobilization. The stability of the immobilized enzyme depends on various factors such as the interaction with the support, binding position, the number of the bonds, conformational freedom, microenvironment, structure of the support, properties of the spacer (charged or neutral, hydrophilic or hydrophobic, size), and immobilization conditions. However, the enhanced stability resulting from immobilization is often ascribed to the intrinsic features of individual immobilization processes. The stability of immobilized enzyme is often determined by an individual stabilization factor or the cumulative effect of several factors. The same immobilization method leads to different stabilities depending on the selected support and immobilization conditions [134]. For example, lipase from *Candida rugosa* was found to be more stable when entrapped in alginate gel than when covalently bound on Eupergit C or encapsulated in a sol–gel matrix [175]. Another striking example is that immobilized glucoamylase entrapped in polyacrylamide gels was found to be more stable than that covalently bound to SP-Sephadex C-50 [176]. Enzyme stabilization by immobilization is currently no longer an exception, because of our increasing understanding of immobilization processes. Thermophilic and hyperthermophilic enzymes are further stabilized by immobilization [177–180], suggesting the additive nature of enzyme stabilization by immobilization.

**Figure 4:**
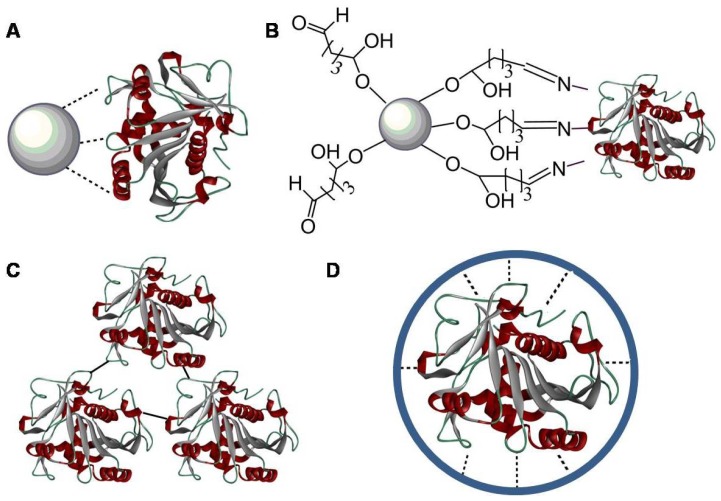
Enzyme stabilization by immobilization introduces additional covalent and non-covalent forces to an external matrix. (**A**) Non-covalent physical adsorption of an enzyme on the nanoparticle; (**B**) covalent binding of an enzyme to the nanoparticle (multipoint attachment); (**C**) covalent crosslinking of enzymes; and (**D**) microencapsulation of an enzyme by a micelle.

Physical adsorption and covalent binding both reduce or avoid enzyme leaching, but binding to a planar surface can lead to decreased stability or even protein denaturation. Crosslinking of enzymes usually increases their stability at the expense of decreased activity. Microencapsulation into micelles or micellar polymers offers the highest potential to significantly increase enzyme lifetime and stop enzyme leaching, although mass transfer problems may occur. The binding of an enzyme to an external organic or inorganic support introduces additional covalent or non-covalent interactions. This additional interaction decreases the structural flexibility of the enzyme and provides rigidity to the immobilized enzyme, which in turn decreases its liability for denaturation. Figure 4 illustrates the different strategies used to immobilize enzymes at support surfaces to enhance stability. Multiple-point attachment (or crosslinking) for stabilization of carrier-bound immobilized enzyme was introduced in the beginning of the 1970s and further explored to design stable carrier-bound immobilized enzymes. The extent of stabilization of immobilized enzyme by multipoint attachment depends on the number of bonds between the enzyme and the support. The characteristics of the support, reactive groups, and immobilization conditions need to be carefully selected to involve the maximum number of enzyme groups in the immobilization. In fact, it has been possible to correlate the enzyme stabilization reached with the number of enzyme-support linkages [181].

Covalent binding of an enzyme to a carrier has the advantage that the enzyme is tightly fixed. This is due to the fact that the formation of multiple covalent bonds between the enzyme and the carrier reduces conformational flexibility and thermal vibrations, thus preventing protein unfolding and denaturation [149,170,182–184]. The amino groups of the enzyme can initiate nucleophilic attack on, for instance, an epoxide or an aldehyde on the support, forming covalent bonds. As in the case of lipase immobilization, shorter spacers confer higher thermal stability, because they restrict enzyme mobility and prevent unfolding. For example, immobilization of β-1,4-glucosidase (BGL) from *Agaricus arvensis* on silicon oxide nanoparticles by covalent binding confers a 288-fold enhancement in half-life over free BGL at 65 °C [149]. α-Amylase was covalently immobilized onto phthaloyl chloride-containing amino group-functionalized glass beads. The immobilized α-amylase exhibited better thermostability than free amylase [185,186]. The covalent immobilization of cellulase and invertase has been shown to improve stability with respect to pH, temperature, and storage, compared to free enzyme in solution [131,187].

Table 3 shows some of the results obtained after immobilizing many different proteins [178,181,188,189]. The stabilization factors are in many cases extremely high (1000- to 10,000-fold) with activity recoveries usually over 60%. Moreover, it should be noted that any other technique employed to obtain a stable enzyme (protein engineering, screening, *etc.*) should be compatible with the stabilization of the enzyme by multipoint covalent attachment, because immobilization will be almost a necessary step for the preparation of an industrial biocatalyst. Thus, enzymes from extremophiles have also been stabilized by multipoint covalent attachment [178,189–191].

### 3.3. Solvent Stability

Enzymes tend to form aggregates in organic solvents and hence tend to be poorly accessible for the substrate. The effects of organic solvents on enzymes are usually detrimental and their adverse influences can be mitigated by using immobilized enzyme preparations. Immobilization of enzymes has improved their activity in organic solvents 100-fold [216,217]. Mesoporous materials have attracted much attention due to their porous structure, which may permit full dispersal of enzyme molecules without the possibility of interacting with any external interface. Moreover, the immobilized enzyme molecules will not be in contact with any external hydrophobic interface [218,219] and cannot inactivate the enzymes immobilized on a porous solid [188]. In the presence of an organic solvent, the immobilized enzymes may be in contact with molecules that are soluble in the aqueous phase, but not with the molecules in the organic phase. Thus, any technique that immobilizes the enzyme inside a porous solid provides operational stability to the enzyme, even without affecting the native structure of the enzyme. However, this stabilization is not universally associated with immobilization. The activity of immobilized enzymes is also influenced by the properties of the support, e.g., the aquaphilicity [220]. For example, laccase immobilized on silica gel was 20-fold and 72-fold more active in diethyl ether than in ethyl acetate and methylene chloride, respectively [221]. Furthermore, overall, the Nylon 66 membrane support resulted in the best laccase activity in these three solvents. This shows that the effect of the support matrix on enzyme activity can be more pronounced than solvent-dependent differences. Wang *et al.* reported the activities of different enzymes immobilized in the molecular hydrogels and their superactivity in toluene relative to unconfined enzymes in water. α-glucosidase from baker’s yeast in organic cosolvents was stabilized by an order of magnitude by immobilization onto macroporous poly(glycidyl methacrylate-co-ethylene glycol dimethacrylate) [222]. Lipase immobilized on Amberlite XAD-7 maintained 100% of its synthetic activity even after 30 hours of incubation in n-hexane, heptanes, or isooctane. In contrast, free lipase showed too low or no hydrolytic activity after incubation in organic solvents [223].

### 3.4. Selectivity

The selectivity of enzymes is nowadays becoming a powerful asset of enzyme-mediated asymmetric synthesis, because of the increasing need of the pharmaceutical industry for optically pure intermediates [163]. The enzyme shows high specificity towards the natural substrate, but this value may be significantly reduced when the enzyme is intended to be used against compounds that are unlike their natural substrate. The selectivity of enzymes includes substrate selectivity, stereoselectivity, and regioselectivity [224]. Modulation of enzyme substrate selectivity has been achieved by selecting the immobilization technique [174]. *Candida rugosa* lipase (CRL) is an important industrial lipase; due to its wide substrate specificity it is successfully utilized in a variety of hydrolysis and esterification reactions. The synthesis of several pharmaceuticals is made possible due to its high stereoselectivity and regioselectivity [186,225]. Genetic engineering has been extensively used to alter enzyme selectivity [226]. However, there are also many interesting examples in which enzyme selectivity has been altered by a variety of immobilization techniques such as covalent bonding, entrapment, and simple adsorption. On many occasions, it has been found that a non-selective enzyme such as chloroperoxidase is transformed into a stereoselective enzyme after immobilization an *S*-selective CRL has also been converted to an *R*-selective CRL by covalent immobilization [227].

Selectivity that can be influenced by the immobilization technique can be classified into two major groups: support-controlled (pore size-controlled and diffusion-controlled) and conformation-controlled (microenvironment-controlled and active site-controlled), on the basis of the effect of the source. For example, the product pattern of controlled pore glass (CPG)-immobilized subtilisin-catalyzed digestion of proteins can be affected by the pore size of the carrier used [228]. Urokinase covalently immobilized on glyoxal agarose exhibited changed selectivity towards glutathione *S*-transferase [198,229]. α-Amylase immobilized on silica [230] or covalently bound to CNBr-activated carboxymethyl cellulose [231] afforded products whose composition differed from that obtained with the native enzyme. This was largely attributed to the fact that the size of the pores where the enzyme molecules are located determines the accessibility of the substrates, depending on their size. Diffusion-controlled enantioselectivity was reported after a study of the enantioselectivity of the lipase CaL-B in transesterification in organic solvents [232]. A relevant example worth mentioning is that simple adsorption of CRL on Celite not only enhanced its stability in acetaldehyde but also enhanced its enantioselectivity up to three-fold [233].

Immobilization may produce some distortion in the enzyme structure, particularly in the active site region. The immobilization of a protein via different regions may therefore confer different rigidities and distort the enzyme in very different ways; it may be even possible to generate different microenvironments around the enzyme with very different physical properties [174]. The enantiopreference of lipases were successfully modulated using the above strategy [227,234–238]. It has been reported that the same lipase immobilized on different supports may exhibit very different enantioselectivities (in some cases even an inversion) in the same experimental conditions. A detailed analysis of the enantiopreferences of lipases has been reported by Mateo *et al.* (2007) [174]. In some cases, a change in experimental conditions can cause opposing effects on different immobilized preparations [227,234,237]. However, these enantioselectivity modulations are examples of random immobilization, which produces alteration even if it is not desirable. The use of this strategy may sometimes result in properties that are less desirable than those of the free enzyme. Therefore, directed immobilization is a powerful tool to modulate enzyme properties.

Directed immobilization involves the selection of the point of attachment through specific interactions between functional groups on the support and the enzyme, based on detailed structural information of the enzyme and the structure of the support. The properties of the immobilized enzyme depend on the modification (the immobilization conditions), the nature of the modifier (nature of support), and the nature of the enzyme (source, purity, and strain). Lipases are the best example and have been most extensively studied for the modulation of enantioselectivity by immobilization. In another successful example reported by Cabrera *et al.* (2009), the stereoselectivity of CaL-B lipase for hydrolysis of racemic 2-*O*-butyryl-2-phenylacetic acid changed both quantitatively (% ee) and qualitatively (*R* or *S* enantiomer product) when bound hydrophobically to different supports, shifting from 99% ee (*S*) on Lewatit to 95% (*R*) on octyl agarose [239]. Yilmaz *et al.* (2011) reported that sporopollenin-based encapsulated lipase in particular had higher conversion and enantioselectivity compared to sol–gel free lipase [186]. These results reveal that sol–gel encapsulated lipase has high enantioselectivity (*E*) and conversion (x) compared with covalently immobilized lipase (*Candida rugosa* lipase). In this study, excellent enantioselectivity (*E* > 400) was noticed for most lipase preparations (*E* = 166 for the free enzyme) with an ee value of 98% for *S*-Naproxen. Recently (Sahin *et al.*, 2009), CRL was encapsulated by polycondensation of tetraethoxysilane and octyltriethoxysilane in the presence of the compounds calix[n]arene, calix[n]–NH, and calix[n]–COOH (*n* = 4, 6, and 8) [240]. For encapsulated CRL-catalyzed hydrolysis of racemic Naproxen methyl ester in aqueous phase/isooctane biphasic medium, the temperature, pH of the aqueous phase, and calix[n]arene-based additives were found to have important effects on the conversion and enantioselectivity. Wang *et al.* (2008) found that the esterase of *Kelbsiella oxytoca* provided a much higher enantiomeric ratio in the hydrolysis of (*R*,*S*)-ethyl mandelate when the enzyme was immobilized on Eupergit C 250 L [241].

With regard to the improvement of enzyme selectivity, there are many exciting examples, as noted above, of immobilized enzymes for which selectivity, e.g., reaction selectivity, substrate selectivity, stereoselectivity, or chemical selectivity, can be affected by the immobilization procedure [242,243], perhaps combined with reaction medium engineering [237]; however, the improvement of enzyme selectivity by immobilization is fundamentally still a new endeavor, lacking guidelines that can be used to guide practical experiments. Nevertheless, with the increasing understanding of the relationship between enzyme selectivity and the structural changes resulting from genetic engineering or other chemical modifications, increasing interest in the improvement of enzyme selectivity by immobilization can be expected in the near future.

### 3.5. Substrate Tolerance

Inhibition of enzymes during the reaction by substrates, reaction products, or components of the bulk medium is major issue in the application of biocatalysts in industrial processes. Depending on the type of inhibition, specific inhibitors tend to interact with the enzyme structure at specific positions to produce the inhibition. Thus, immobilization may reduce inhibition based on the type of inhibition mechanism in different situations. First, when the inhibitor acts allosterically, interacting with the protein in a place different from the active center, the allosteric site of enzyme may be blocked during the course of the immobilization procedure, significantly reducing the inhibition caused by this mechanism. Second, when the inhibitor interacts with the active site of the protein, immobilization may slightly distort the enzyme active site, and in some cases a higher distortion of the enzyme may be achieved in the inhibitor binding site than in the substrate binding site. This is more likely if the substrate is larger than the inhibitor and interacts with a larger number of groups in the enzyme.

A lactase from *Kluyveromyces lactis* shows competitive inhibition by galactose (χ = 45 mM) and non-competitive by glucose (χ = 750 mM) [244]; upon immobilization of this lactase, the inhibition constant for galactose was improved from 45 mM to 40 M. Similar enhancement was obtained in the case of lactase from *Thermus sp.* by galactose (χ = 3.1 mM) and non-competitive inhibition by glucose (χ was 50 mM) [245]. The immobilization of lactase on different supports permitted the screening of the preparation, where the χ by glucose was increased by a two-fold factor, while the competitive χ by galactose was increased by a four-fold factor [245]. Another example of reduced substrate inhibition and increased activity upon immobilization on porous cellulose beads has been reported for invertase from *Candida utilis* [132].

### 3.6. Multi-Step Reactions

Co-immobilized multi-enzymatic systems are increasingly driven by economic and environmental constraints that also permit multi-enzyme reactions through artificial enzymatic cascade processes through compartmentalization of the individual catalysts [217]. For example, Brazeau *et al.* (2008) have developed a multi-enzyme pathway, immobilized on Eupergit C, for the synthesis of the monatin [246]. Co-immobilization of three cellulases on Au-doped magnetic silica nanoparticles for the degradation of cellulose has been reported by Cho *et al.* [247]. The simultaneous co-immobilized three cystein-tagged cellulases, including endo-glucanase (EGIVCBDII), exo-glucanase (CBHII), and β-glucosidase (BglB) co-immobilized on AuNP (gold nanoparticles) and Au-MSNP (gold-doped magnetic silica nanoparticles) was successfully demonstrated for the production of cellobiose and glucose. Co-immobilization of coupled enzyme systems can enhance activity and stability [248] such as where nitrobenzene nitroreductase and glucose-6-phosphate dehydrogenase were co-encapsulated in silica particles, wherein the G6PD allowed regeneration of NADPH. St. Clair *et al.* (2000) also demonstrated that the CLEC could also retain cofactors for redox reactions [249]. As such, co-immobilization provides benefits that span numerous biotechnological applications, from biosensing of molecules to cofactor recycling and to combination of multiple biocatalysts for the synthesis of valuable products.

Selective compartmentalization of enzymes during immobilization could provide advantages. An interesting example of multi-enzyme compartmentalization was reported by Dongen *et al.* [250]. The co-polymer of isocyanopeptides and styrene was used to form porous polymersomes immobilized with horseradish peroxidize, CaL-B and glucose oxidase anchored to the membrane surface, bilayer membrane, and in the polymersome lumen, respectively. The three enzymes were able to perform a three sequential steps reaction using glucose acetate as the initial substrate (which was subsequently deacetylated by the lipase and oxidized by the glucose oxidase), yielding peroxide that was subsequently used by the peroxidize to oxidize ABTS [2,20-azinobis (3-ethylbenzothiazoline-6- sulfonic acid)]. Similar uses of compartmentalization and multiple enzymes (laccase and lipase) have been achieved using Spherezymes [251]. α-Amylase, cellulase, protease, and lipase have been immobilized individually on various supports. However, Pundir *et al.* (2012) reported the covalent coimmobilization of commercial α-amylase, cellulase, protease, and lipase onto the inner side of a plastic beaker and bristles of a plastic brush, their properties, and use in removal of stain from cloth [252]. Similarly, commercial lipase, glycerol kinase (GK), glycerol-3-phosphate oxidase (GPO) and peroxidase were co-immobilized covalently on to arylamine glass beads [253]. Starch-converting enzymes, α-amylase and glucoamylase, were immobilized on surface-modified carriers using a co-immobilized, as well as a single system. The co-enzymes immobilized on hydrophilic silica gel and DEAE-cellulose entrapped in alginate beads exhibited 92.3 and 88.9% of the remaining activity even after 10 times of reuse [254]. Co-immobilized enzymes have been used extensively in expensive commercial and biological approaches in recent years to develop more economic and environmentally friendly processes.

### 3.7. Advances in Enzyme Immobilization

Recent advancements in nano and hybrid technology have made various materials more affordable hosts for enzyme immobilization. As a result, various nanostructured materials based on combined organic and inorganic species have received attention as enzyme immobilizing supports due to their intrinsic large surface areas and physicochemical properties such as pore size, hydrophilic/hydrophobic balance, aquaphilicity and surface chemistry [255]. Large surface area and controlled pore size often result in improved enzyme loading, which increases enzyme activity per unit mass or volume, compared to that of conventional materials [256]. Nanostructured materials with ability to control size and shape enables better interaction with the enzyme, increases immobilization efficiency, and enhances the long-term storage and recycling stability of the enzyme [257]. In addition, these organic, inorganic or hybrid materials may provide specific features such as enhanced strength, elasticity, plasticity, and chemical bonding in an appropriate microenvironment. Various organic or inorganic nanomaterials such as multiwall carbon nanotubes (MWCNTs), magnetic nanoparticles, silica nanoparticles, and quantum dots have been exploited to generate hybrid composite nanofibers with additional physical properties and mechanical stability [258,259].

Magnetite mesoporous silica hybrid support was fabricated by depositing magnetite and MCM-41 nanoparticles onto polystyrene beads using the layer-by-layer (LBL) method. The incorporation of magnetite gives an additional magnetic property to the hollow mesoporous silica shells to improve the enzyme immobilization. Magnetic/silica nanoparticles with a core of magnetic clusters were applied to stabilization of His-tagged *Bacillus stearothermopilus* L1 lipase [260]. Xia’s group reported on encapsulation of enzymes in nanofibers by direct coelectrospinning [261].

#### 3.7.1. New Technology for Enzyme Immobilization

In the last decade, more attention has been paid to the regulation of the microenvironment for the enzyme on a support surface in order to obtain significant stabilization of the immobilized enzyme and development of new support. Unfortunately, there have been few studies in enzyme immobilization that have focused on mimicking the environment of a cell to improve the properties of the enzyme *in vitro*. Moreover, many attempts have been focused on the multipoint covalent attachment of proteins on supports, which has been reported to clearly increase the thermal stability of immobilized enzymes. However, the stabilizing effect increases with the number of covalent bonds between enzymes and the support until some critical value (a limit) is achieved, and a further increase in the number of bonds does not result in further stabilization [262]. Excess activated groups present on the support surface would increase the possibility of enzyme deactivation due to their subsequent slow reaction with the enzyme [168].

##### 3.7.1.1. Microwave Irradiation

Recently, controlled microwave irradiation has been shown to dramatically accelerate chemical reactions and reduce reaction times required for immobilization [263]. It is thought that microwave irradiation provides an additional driving force for mass transport and accelerates the mass transfer [264]. Microwave irradiation has been used to simplify and improve the reaction conditions for many classic organic reactions. Reactions performed under microwave irradiation proceed faster, are cleaner, present much better yields and are more reproducible than those performed under conventional conditions. Microwave assisted heterogeneous oxidation and reduction reactions have received attention in recent years. Varma *et al.* have demonstrated that the reaction rate of oxidation and reduction reaction under microwave irradiation could be greatly enhanced under microwave irradiation than that under conventional heating [265,266].

Wang *et al.* have immobilized papain and penicillin acylase in mesocellular siliceous foams (MCFs) by using the microwave irradiation technology [267,268]. 80 and 140 seconds were enough for immobilization of papain and penicillin acylase, respectively. The maximum loading of papain reached 984.1 mg/g, 1.26 times of that obtained from the conventional method which was non-microwave-assisted. The activities of microwave-assisted immobilized papain and penicillin acylase were 1.86 and 1.39 times of those with conventional method. Immobilization of lipases and horseradish peroxidase under microwave-assisted method were reported [269].

##### 3.7.1.2. Photoimmobilization Technology

The protein molecules (e.g., horseradish peroxidase or glucose oxidase), when exposed to ultraviolet (UV) light at 365 nm along with a photoreactive polymer, were immobilized through covalent bonding by the highly reactive nitrene of the polymer in about 10 to 20 min [270]. The photoreactive polymer under UV light generates highly reactive nitrene, which has a property of inserting into C–H bond and is capable of binding with the biomolecule thus serving important for the immobilization of biomolecules irrespective of their functional groups. However, Kumar and Nahar (2007) reported that horseradish peroxidase and glucose oxidase showed efficient immobilization when placed on the photoreactive cellulose membranes and exposed to sunlight [271]. No detectable increase in immobilization was observed beyond 21,625 lux, which was considered as the sunlight intensity required for optimum immobilization. Thus, sunlight could be a very good alternative compared to 365 nm UV light for photoimmobilization. Therefore, photoimmobilization is a green technique and suitable for large-scale, as well as small-scale, immobilization.

##### 3.7.1.3. Enzymatic Immobilization of Enzyme

The use of green chemistry rather than harsh chemicals is one of the main goals in enzyme industries to avoid the partial denaturation of enzyme protein. Enzyme-assisted immobilization of the enzyme is an emerging and novel technology to fabricate solid protein formulations [272,273]. Tanaka *et al.* (2007) reported the immobilization of enhanced green fluorescent protein (EGFP) and glutathione *S*-transferase (GST) onto the casein-coated polystyrene surface as model proteins. EGFP and GST were tagged with a neutral Gln-donor substrate peptide for MTG (Leu-Leu-Gln-Gly, LLQG-tag) at their *C*-terminus. This strategy has been also applied to immobilize Luciferase (Luc) and GST ybbR-fusion proteins and thioredoxin fusion proteins [273].

##### 3.7.1.4. Controlled Immobilization of Enzyme onto Porous Materials

Protein distribution (heterogeneous or homogeneous) across a porous material depends on whether the immobilization rate is controlled by either protein/support attachment or mass transference. Bolivar *et al.* (2011) described different strategies to control the distribution of fluorescent proteins by altering their immobilization rates under low protein loads conditions focusing on the chemical nature of protein-support attachment [274]. The control of protein immobilization by modulation of apparent immobilization rate was extended to the immobilization of two fluorescent proteins onto the same carrier, creating four different distribution patterns. These tailor-made distributions of two proteins inside one single porous carrier are pioneering in immobilization technology. One of these groups is highly reactive under alkaline conditions, thereby promoting intense covalent protein-support attachments, whereas the other group reversibly immobilizes the protein under mild pH conditions (pH 6–8). Another interesting approach to control the distribution of protein is the immobilization of multi-enzyme systems on the same solid surface that is activated by different reactive groups. Recently, Rocha-Martín *et al.* (2012) reported the co-immobilization of three bio-redox orthogonal cascades that involved a main and a recycling dehydrogenase onto a heterogeneously activated agarose-type support [275]. The agarose-type support was heterogeneously activated with glyoxyl groups and metal chelates to enable the immobilization of proteins through two different techniques.

#### 3.7.2. Recommendation for the Future of Immobilization Technology

At present, a vast number of methods for immobilization of enzymes are available. Unfortunately, there is no universal immobilization method, nor a single preferred method of enzyme immobilization. Immobilization method or the choice of support differs from enzyme to enzyme, from application to application and from carrier to carrier. Cao (2005), in his book *Carrier Bound Immobilized Enzymes*, suggested that the major problem in enzyme immobilization is not only the selection of the right carrier for the enzyme immobilization, but it is how to design the performance of the immobilized enzyme [168]. In the future, information derived from protein sequences, 3D-structures, reaction mechanism, and working environment should be further combined with the properties of supports and immobilization methods in order to produce the immobilized enzyme with enhanced properties for a biochemical application. As methods for the immobilization of enzymes continue to improve and become commercially widespread, the cascade enzymatic reaction and *in vitro* synthetic biology, multi-enzyme immobilization will be one of next goals [276–278]. In addition, with the growing attention paid to the use of *in silico* technology in various fields of sciences, including protein engineering, the *in silico* model can be designed to validate the probability of success and the efficiency of the immobilization process before starting the immobilization.

### 3.8. Integration of Different Techniques

There are many reports showing that the enzyme properties may be directly improved by: chemical or genetic modification of enzymes, screening of the most suitable enzyme, and immobilization of enzymes. An excellent example of the coupling of engineering and immobilization is demonstrated by the variants of formate dehydrogenase whereby *Candida boidinii* preserved 4.4-fold higher activity after entrapment in polyacrylamide gels than the wild-type enzyme [159]. Godoy *et al.* (2011) reported the site-directed immobilization/rigidification of genetically modified enzymes through multipoint covalent attachment on bifunctional disulfide-glyoxyl supports [279]. Genetically engineered lipase 2 from *Geobacillus thermocatenulatus* and penicillin G acylase from *E. coli* were immobilized and uniquely rigidified by a multipoint covalent attachment onto tailor-made bifunctional disulfide-glyoxyl supports. Therefore, the use of coupled strategy (protein engineering and immobilization) seems to improve the properties of the final immobilized biocatalyst, and, moreover, modulates multiple properties of enzymes for industrial application.

## 4. Conclusions

In this review, we have shown some examples of where the modulation of biochemical and physical properties of engineered and/or immobilized enzymes has been permitted to solve problems such as stability, selectivity, and substrate or solvent tolerance of enzymes. It is clear from this report that protein engineering or immobilization individually cannot make an ideal catalyst for industrial processes. Until now, there has been no universal method to modulate a particular property of an enzyme. Therefore, the combination of protein engineering and immobilization of enzymes seems to be a powerful tool to greatly improve a number of industrial processes, and more effort may be expected in the coming years in this regard. When properly designed, the immobilization of enzymes in combination with protein engineering techniques can be a very successful strategy to improve enzymes of industrial significance.

## Figures and Tables

**Table 1:** List of the enzymes engineered by protein engineering.

Enzyme	Organism	Improved property	Method	Application	Reference
Hydantoinase	*Arthrobacter* sp.	Enantioselective hydantoinase and 5-fold more productivity	Saturation mutagenesis, screening	Production of l-Met (l-amino acids)	[7]

Cyclodextrin glucanotransferase	*Bacillus stearothermophilus* ET1	Modulation of cyclizing activity and thermostability	Site-directed mutagenesis	Bread industry	[8]

Lipase B	*Candida antarctica*	20-fold increase in half-life at 70 °C	epPCR	Resolution and desymmetrization of compound	[9]

Tagatose-1,6-Bisphosphate aldolase	*E. coli*	80-fold improvement in k_cat_/K_m_ and 100-fold change in stereospecificity	DNA shuffling and screening	Efficient syntheses of complex stereoisomeric products	[10]

Xylose isomerase	*Thermotoga neapolitana*	High activity on glucose at low temperature and low pH	Random Mutagenesis and screening	Used in preparation of high fructose syrup	[11]

Amylosucrase	*Neisseria polysaccharea*	5-fold increased activity	Random mutagenesis, gene shuffling, and directed evolution	Synthesis or the modification of polysaccharides	[12]

Galactose oxidase	*F. graminearum*	3.4–4.4 fold greater V_max_/K_m_ and increased specificity	epPCR and screening	Derivatization of guar gum	[13]

Fructose bisphosphate aldolase	*E. coli*	Increased thermostablity and stability to treatment with organic solvent	DNA shuffling	Use in organic synthesis	[14]

1,3-1,4-α-d-glucanase	*Fibrobacter succinogenes*	3–4-fold increase in the turnover rate (k)	PCR-based gene truncation	Beer industry	[15]

Lipase	*P. aeruginosa*	2-fold increase in amidase activity	Random mutagenesis and screening	Understanding lipase inability to hydrolyze amides	[16]

Protease BYA	*Bacillus* sp. Y	Specific activity1.5-fold higher	Site-directed mutagenesis	Detergents products	[17]

*p*-Hydroxybenzoate hydroxylase	*Pseudomonas fluorescens* NBRC 14160	Activity, reaction specificity, and thermal stability	Combinatorial mutagenesis	Degrading various aromatic compounds in the environment	[18]

Endo-1,4-β-xylanase II	*Trichoderma reesei*	Increased alkali stability	Site-directed mutagenesis	Sulfate pulp bleaching	[19]

Xylose isomerase	*Thermotoga neapolitana*	2.3-fold increases in catalytic efficiency	Random mutagenesis	Production of high fructose corn syrup	[11]

α-Amylase	*Bacillus* sp. TS-25	10 °C enhancement in thermal stability	Directed evolution	Baking industry	[20]
Xylanase		Tm improved by 25 °C	Gene site-saturation mutagenesis	Degradation of hemicellulose	[21]
Fructosyl peptide oxidase	*Coniochaeta* sp	79.8-fold enhanced thermostability	Directed evolution and site-directed mutagenesis	Clinical diagnosis	[22]
Endo-β-1,4-xylanase	*Bacillus subtilis*	Acid stability	Rational protein engineering	Degradation of hemicellulose	[23]
Subtilase	*Bacillus* sp.	6-fold increase in caseinolytic activity at 15–25 °C	Directed evolution and site-directed mutagenesis	Detergent additives and food processing	[24]
CotA laccase	*B. subtilis*	120-fold more specific for ABTS	Directed evolution	Catalyze oxidation of polyphenols	[25]
Pyranose 2-oxidase	*Trametes multicolor*	Altered substrate selectivity for d-galactose, d-glucose	Semi-rational enzyme engineering approach	Food industry	[26]
Xylanase XT6	*Geobacillus stearothermophilus*	52-fold enhancement in thermostability; increased catalytic efficiency	Directed evolution and site-directed mutagenesis	Degradation of hemicellulose	[27]
Lipase	*Bacillus pumilus*	Thermostability and 4-fold increase in k_cat_	Site-directed mutagenesis	Chemical, food, leather and detergent industries	[28]
Bgl-licMB	*Bacillus amyloliquefaciens* (Bgl) *a*nd *Clostridium thermocellum* (licMB)	2.7 and 20-fold higher k_cat_/K_m_ than that of the parental Bgl and licMB, respectively	Splicing-by-overlap extension	Brewing and animal-feed industries	[29]
β-agarase AgaA	*Zobellia galactanivorans*	Catalytic activity and thermostability	Site-directed mutagenesis	Production of functional neo-agarooligosaccharides	[30]
Prolidase	*Pyrococcus horikoshii*	Thermostability	Random mutagenesis	Detoxification of organophosphorus nerve agents	[31]
Lipases	*Geobacillus* sp. NTU 03	79.4-fold increment in activity; 6.3–79-fold enhanced thermostability	Error-prone PCR and site-saturation mutagenesis	Transesterification	[32]
Xylanase	*Hypocrea jecorina*	Thermostability	Look-through mutagenesis (LTMTM) and combinatorial beneficial mutagenesis (CBMTM)	Degradation of hemicellulose	[33]
Amylase	*Bacillus* sp. US149	Thermostability	Site-directed mutagenesis	Bread industry	[34]
Cholesterol oxidase	*Brevibacterium* sp.	Thermostability and enzymatic activity	Site-directed mutagenesis	Detection and conversion of cholesterol	[35]
Lipase B	*Candida antarctica*	Enhancement of thermostability	Molecular dynamics (MD) simulation and site-directed mutagenesis	Detergent industries	[36]
Laccase	*Bacillus* HR03	3-fold improved k_cat_ and thermostability	Directed mutagenesis	Catalyze oxidation of polyphenols, and polyamines	[37]
d-psicose 3-epimerase	*Agrobacterium tumefaciens*	Thermostability	Random and site-directed mutagenesis	Industrial producer of d-psicose	[38]
1,3-1,4-β-d-glucanase	*Fibrobacter succinogenes*	Thermostability and specific activity	Rational mutagenesis	Widely used as a feed additive	[39]
α-Amylase	*Bacillus licheniformis*	Acid stability	Direct evolution	Starch hydrolysis	[40]
Alkaline amylase	*Alkalimonas amylolytica*	Oxidative stability	Site-directed mutagenesis	Detergent and textile industries	[41]
Endoglucanase	*Thermoascus aurantiacus*	4-fold increase in k_cat_ and 2.5-fold improvement in hydrolytic activity on cellulosic substrates	Site-directed mutagenesis	Bioethanol production	[42]
d-glucose 1-dehydrogenase isozymes	*Bacillus megaterium*	Substrate specificity	Site-directed mutagenesis	Measurements of blood glucose level	[43]
Glycerol dehydratase	*Klebsiella pneumoniae*	2-fold pH stability; enhanced specific activity	Rational design	Synthesis of 1,3-Propanediol	[44]
Cyclodextrin Glucanotransferase	*Bacillus* sp. G1	Enhancement of thermostability	Rational mutagenesis	Starch is converted into cyclodextrins	[45]
Cellobiose phosphorylase	*Clostridium thermocellum*	Enhancement of thermostability	Combined rational and random approaches	Phosphorolysis of cellobiose	[46]
Superoxide dismutase	*Potentilla atrosanguinea*	Thermostability	Site-directed mutagenesis	Scavenging of O_2_^−^	[47]
Endoglucanase Cel8A	*Clostridium thermocellum*	Thermostability	Consensus-guided mutagenesis	Conversion of cellulosic biomass to biofuels	[48]
Endo β-glucanase EgI499	*Bacillus subtilis* JA18	Increase in half life from 10 to 29 mins at 65 °C	Deletion of *C*-terminal region	Animal feed production	[49]
Pyranose 2-oxidase	*Trametes multicolor*	Increase half life from 7.7 min to 10 h (at 60 °C)	Designed triple mutant	Food industry	[50]
Xylanase XT6	*Geobacillus stearothermophilus*	52× increase in thermal stability, k_opt_ increase by 10 °C, catalytic efficiency increase by 90%	Directed evolution and site-directed mutagenesis	Biobleaching	[27]
Tyrosine phenol-lyase	*Symbiobacterium toebi*	Improved thermal stability and activity (Increase in T_m_ up to 11.2 °C)	Directed evolution (random mutagenesis, reassembly and activity screening)	Industrial production of l-tyrosine and its derivatives	[51]
Phytase	*Penicilium* sp.	Increased thermal stability	Random mutation and selection	Feed additives	[52]
l-Asparaginase	*Erwinia carotovora*	Increase in half-life from 2.7 to 159.7 h	*In vitro* directed evolution	Therapeutic agent	[53]
Endoglucanase CelA	*Clostridium thermocellum*	10-fold increase in half-life of inactivation at 86 °C	Saturation mutagenesis	Bioconversion of cellulosic biomass	[54]
β-glucosidase BglC	*Thermobifida fusca*	Increase in half-life from 12 to 1244 min	Family shuffling, site saturation, and site-directed mutagenesis	Bioconversion of cellulosic biomass	[55]
Phospholipase D	*Streptomyces*	Improved thermal stability and activity	Semi-rational, site-specific saturation mutagenesis	Phosphatidylinositol synthesis	[56]
β-glucosidase	*Trichoderma reesei*	Enhanced k_cat_/K_m_ and k_cat_ values by 5.3- and 6.9-fold	Site-directed mutagenesis	Hydrolysis of cellobiose and cellodextrins	[57]
Lipases		144-fold enhanced thermostability	Error prone PCR	Synthesis and hydrolysis of long chain fatty acids	[58]
Laccase	*Pycnoporus cinnabarinus*	8000-fold increase in k_cat_/K_m_	Directed evolution and semi-rational engineering	Lignocellulose biorefineries, organic synthesis, and bioelectrocatalysis	[59]
Feruloyl esterase A	*Aspergillus niger*	Increase in half-life from 15 to >4000 min	Random and site-directed mutagenesis	Degradation of lignocellulose	[60]

**Table 2:** Examples of immobilized enzymes with enhanced activity.

Enzyme	Applications	Kinetic parameters	Reference
α-Chymotrypsin	Proteolysis (cleave Peptide amide bonds)	Immobilized enzyme: K_m_ = 31.7 μM, k_cat_ = 20.0 s^−1^; soluble enzyme: K_m_ = 47.8 μM, k_cat_ = 17.8 s^−1^	[141]

β-glucosidase	Lignocellulose hydrolysis	Immobilized enzyme: K_m_ = 10.8 mM, V_max_ = 2430 μmol·min^−1^·mg^−1^; soluble enzyme: K_m_ = 1.1 mM, V_max_ = 296 μmol·min^−1^·mg^−1^	[142]

Glucose oxidase	Estimation of glucose level up to 300 mg·mL^−1^	Immobilized enzyme: K_m_ = 3.74 mM, soluble enzyme = 5.85 mM	[143]
Diastase	Starch hydrolysis	Immobilized enzyme: K_m_ = 8414 mM, V_max_ = 4.92 μmol min^−1^ mg^−1^; soluble enzyme: K_m_ = 10,176 mM, V_max_ = 2.71 μmol min^−1^ mg^−1^	[144]

β-galactosidase	GOS synthesis	Immobilized enzyme: k_1_ = 1.41 h^−1^; soluble enzyme: k_1_ = 1.16 h^−1^	[145]

Keratinase	Synthesis of keratin	Immobilized enzyme: specific activity = 129.0 U·mg^−1^; soluble enzyme: specific activity = 37 U·mg^−1^	[146]

Horseradish peroxidase		Immobilized enzyme: K_m_ = 0.8 mM, V_max_ = 0.72 μmol min^−1^ mg^−1^; soluble enzyme: K_m_ = 0.43 mM, V_max_ = 0.35 μmol min^−1^ mg^−1^	[147]

Glucose oxidase	Estimation of glucose level	Immobilized enzyme: K_m_ = 2.7 mM, V_max_ = 28.6 U·μg^−1^; soluble enzyme: K_m_ = 9 mM, V_max_ = 6.2 μmol·min^−1^ mg^−1^	[148]

β-1,4-glucosidase (*Agaricus arvensis*)	Lignocellulose hydrolysis	Immobilized enzyme: K_m_ = 3.8 mM, V_max_ = 3,347 μmol min^−1^ mg^−1^; soluble enzyme: K_m_ = 2.5 mM, V_max_ = 3,028 μmol min^−1^ mg^−1^	[149]

l-arabinose isomerase (*B. licheniformis*)		Immobilized enzyme: K_m_ = 352 mM, V_max_ = 326 μmol min^−1^ mg^−1^; soluble enzyme: K_m_ = 369 mM, V_max_ = 232 μmol min^−1^ mg^−1^	[150]

Diastase α-amylase	Hydrolyzing soluble starch	Immobilized enzyme: K_m_ = 10.3 mg/mL; V_max_ = 4.36 μmol min^−1^ mg^−1^ mg^−1^; soluble enzyme: K_m_ = 8.85 mg mL^−1^; V_max_ = 2.81 μmol·min^−1^·mg^−1^	[151]

Cellobiase	Bioethanol production	Immobilized enzyme: K_m_ = 0.30 mM, V_max_ = 6.77 μM min^−1^; soluble enzyme: K_m_ = 2.48 mM, V_max_ = 2.38 μM min^−1^	[152]

Laccase	Bioremediation of environmental pollutants	Immobilized enzyme: K_m_ (10^−2^ mM) = 10.7, V_max_ (10^−2^ mM min^−1^) = 14.0; soluble enzyme: K_m_ (10^−2^ mM) = 5.69, V_max_ (10^−2^ mM min^−1^) = 7.7	[153]

Keratinase	Synthesis of keratin	Immobilized enzyme: specific activity = 129 U mg^−1^; soluble enzyme: specific activity = 37 U mg^−1^	[146]

Raw starch digesting amylases	Starch hydrolysis	Immobilized enzyme: K_m_ (10^−1^) = 3.8 mg mL^−1^, V_max_ = 27.3 U·mg^−1^; soluble enzyme: K_m_ (10^−1^) = 3.5 mg mL^−1^, V_max_ = 23.8 U·mg^−1^	[154]

Aldolase		Immobilized enzyme: K_m_ = 0.10 mM; k_cat_/K_m_ = 584 min^−1^·mM^−1^, soluble enzyme K_m_ = 0.12 mM; k_cat_/K_m_ = 540 min^−1^·mM^−1^	[155]

α-galactosidase (*Aspergillus terreus*_GR_)	Animal feed	Immobilized enzyme: K_m_ =1.40 mM, V_max_ =20.16 U mL^−1^; soluble enzyme: K_m_ = 4.2 mM, V_max_ =16.33 U·mL^−1^	[156]

Laccase	Textile wastewater treatment	Immobilized enzyme: K_m_ = 0.0717 mM, V_max_ = 0.247 mM·min^−1^; soluble enzyme: K_m_ = 0.0044 mM, V_max_ = 0.024 mM·min^−1^	[157]

Papain	Food, pharmaceutical, leather, cosmetic, and textile industries	Immobilized enzyme: K_m_ = 0.308 × 10^5^ g·mL^−1^; V_max_ = 5.4 g mL^−1^ s^−1^; soluble enzyme: K_m_ = 0.236 × 10^5^ g·mL^−1^; V_max_ = 4.08 g·mL^−1^·s^−1^	[158]

**Table 3:** Examples of enzyme stabilization by immobilization.

Enzyme	Recovered activity (%)	Stabilization factor a	Reference
Lipase (*C. rugosa*)	50	150 a	[192]
Penicillin G acylase (*E. coli*)	70	8000 a	[193]
Chymotrypsin	70	60,000 a	[194]
Penicillin G acylase (*K. citrophila*)	70	7000 a	[195]
Esterase (*B. stearothermophilus*)	70	1000 a	[178]
Thermolysin (*B. thermoproteolyticus*)	100	100 a	[191]
Cholesterol oxidase	nd	2.5 (50 °C)	[196]
Alcalase	54	500	[197]
Urokinase	80	10	[198]
α-Amylase (*B. licheniformis*)	nd	2 (70 °C)	[199]
Invertase	nd	2 (70 °C)	[200]
Dextransucrase (*L. mesenteriodes*)	nd	40 (30 °C)	[201]
Formate dehydrogenase (*Pseudomonas* sp. 101)	50	>5000 a	[188]
Alcohol dehydrogenase (H. Liver)	90	>3000	[202]
Cyclodextrin glycosyltransferase (*B. circulans*)	70	>100	[203]
Formate dehydrogenase (*C. boidini*)	15	150 a	[204]
Laccase (*Rhus vernicifera*)	80	6.4 (65 °C)	[205]
Xylitol dehydrogenase (*Rhizobium etli*)	92	2.2 (60 °C)	[206]
Laccase (*Trametes versicolor*)	69	2.5 (45 °C)	[207]
β-1,4-glucosidase (*Agaricus arvensis*)	158	288 (65 °C)	[149]
Cellulase (*Trichoderma viride*)	nd	2 (55 °C)	[208]
β-Galactosidase	nd	17 (55 °C)	[209]
Lipase G (*Penicillium camembertii*)	nd	1.7 (40 °C)	[210]
Phytases (*Aspergillus niger*)	66	7 (60 °C)	[211]
Phytases (*Escherichia coli*)	74	9.7 (60 °C)	[211]
L-arabinose isomerase (*Bacillus licheniformis*)	145	137.5 (50 °C)	[150]
Protease (*Aspergillus oryzea*)	85	3.5 (70 °C)	[212]
Papain	40	4.2 (70 °C)	[213]
Cellobiase	284	1.2 (60 °C)	[152]
Invertase	NR	3.5 (55 °C)	[214]
α-Amylase (*Bacillus amyloliquifaciens* TSWK1-1)	91	3.75 (60 °C)	[215]
α-Galactosidase (*Aspergillus terreus*_GR_)	74	3.5 (65 °C)	[156]

a Compared with one-point immobilized enzymes.
